# Acquired melanocytic naevus phenotypes and *MC1R* genotypes in Han Chinese: a cross-sectional study

**DOI:** 10.7717/peerj.4168

**Published:** 2017-12-20

**Authors:** Xiaohong Li, Katie J. Lee, David L. Duffy, Dandan Xu, Madhur Eshwar Rao Basude, Ying Hu, Hang Zhang, Kasturee Jagirdar, H. Peter Soyer, Huiting Dong, Richard A. Sturm

**Affiliations:** 1 Department of Dermatology, the First Affiliated Hospital of Zhengzhou University, Zhengzhou University, Zhengzhou, Henan, China; 2 Key-Disciplines Laboratory, Clinical-Medicine Henan, Zhengzhou, Henan, China; 3 Dermatology Research Centre, The University of Queensland Diamantina Institute, Brisbane, Queensland, Australia; 4 QIMR Berghofer Medical Research Institute, Brisbane, Queensland, Australia; 5 Department of Dermatology, Huaihe Hospital of Henan University, Kaifeng, Henan, China; 6 Department of Dermatology, Vinayaka Missions Medical College and Hospital, Karaikal, Puducherry, India; 7 Department of Dermatology, Central Hospital of Zhengzhou, Zhengzhou, Henan, China

**Keywords:** Acquired melanocytic naevi, Melanocortin 1 receptor, Dermoscopy, Han ethnic group

## Abstract

**Background:**

Early detection and treatment are the most important elements in reducing the incidence of melanoma deaths. Acquired melanocytic naevi (AMN) are well-known precursors of melanoma but most of our knowledge on the clinico-dermoscopic phenotypes of AMN is based on studies in European-background populations, particularly American and Australian populations. There has been little research in Chinese Han populations on clinico-dermoscopic variability of naevi or how naevi are affected by melanoma-linked variants of the melanocortin 1 receptor (*MC1R*) gene.

**Methods:**

Clinical and dermoscopic features of 448 AMN in 115 patients from the Han ethnic group in mainland China were described. Germline polymorphisms in *MC1R* were determined for 98 of these patients.

**Results:**

AMN were predominantly found on the head and neck. Dermoscopic patterns observed were nonspecific, reticular, globular, and parallel furrow, with most AMN having a nonspecific pattern. There were no associations between *MC1R* polymorphisms and clinical or dermoscopic features of AMN.

**Discussion:**

Our results provide evidence that AMN in the Han population in China have similar dermoscopic patterns to those in European populations, but are present in much lower numbers. As there were no associations between clinical or dermoscopic features of AMN and *MC1R* polymorphisms, further studies should focus on candidate gene associations with AMN features and the risk of melanoma, with larger sample sizes and comparisons to AMN in other populations.

## Introduction

Acquired melanocytic naevi (AMN) are common benign neoplasms, which can also be a precursor to melanoma; however, AMN have mostly been studied in European-background populations, particularly American and Australian populations. The prevalence, clinical characteristics and dermoscopic features of AMN in ethnic groups with darker skin tones are different than in pale-skinned populations. Skin tones are generally classified according to Fitzpatrick skin phototypes, a scale of I–VI based on the amount of melanin in the skin and the skin’s reaction to UV exposure (Fitzpatrick, 1988). AMN in people with Fitzpatrick skin type V/VI (V: brown skin, rarely burns, tans darkly easily; VI: dark brown or black skin, never burns, always tans darkly) usually have a brown colour with a tendency toward reticular pattern and hyperpigmentation, while AMN in skin type I/II (I: pale or milky skin, always burns, never tans; II: fair skin, burns easily, tans poorly) usually have a light brown colour and a tendency toward structureless pattern and multiple areas of hypopigmentation (Tuma et al., 2015). These characteristics are generally observed with dermoscopy, a well-established non-invasive tool for the differential diagnosis of melanocytic lesions.

The continuous range of skin pigmentation between and within different ethnic groups are the result of interactive and quantitative effects of many polymorphisms in several major genes. Constitutive skin pigmentation, the skin’s baseline melanin production (Slominski et al., 2004; Slominski, Zmijewski & Pawelek, 2012), is regulated by the melanocortin 1 receptor gene *MC1R*, *ASIP*, *TYR*, *HERC2/OCA2*, *SLC45A2* and *SLC24A5*. Increased pigmentation due to UV exposure is heavily influenced by *MC1R*. UV-induced DNA damage causes upregulation of proopiomelanocortin (POMC), a precursor ligand that is cleaved to form *α*-MSH and ACTH ligands, which bind to MC1R and lead to activation of the cAMP-PKA-CREB pathway to produce and deliver extra melanin to the skin (Slominski et al., 2000; Slominski et al., 2013). Downstream activation of microphthalmia transcription factor (*MITF*) and *IRF4* activation lead to the transcription of the melanogenic genes *TYR*, *TYRP1* and *DCT*.

MC1R is highly polymorphic and several polymorphisms are non-functional or partially-functional; they have been associated with pigment phenotype and skin cancers in many recent studies, and play an important role in an individual’s melanoma risk (Sturm et al., 2003; Quint et al., 2012; Yamaguchi et al., 2012). Several polymorphisms, referred to as *R* alleles, are associated with red hair, pale skin and an increased risk of melanoma. Several other polymorphisms, categorized as *r*, are associated with a moderately increased risk of melanoma; some are weakly associated with the red hair phenotype in European populations, and others are not associated with red hair colour. Functionally, *R* alleles are either intracellularly retained or expressed on the cell surface with impaired G-protein coupling, substantially reducing *α*-MSH’s ability to activate the cAMP pathway and ultimately impairing the cell’s ability of produce melanin. In contrast, *r* alleles have a milder functional impairment, usually in proportion to the level of cell surface receptor expression. One exception to this is the *r* allele V92M, which has similar or higher cAMP activation to the wild-type allele; in European populations, this allele is associated with darker skin and hair in homozygous individuals, but lighter skin and hair in heterozygous individuals (Nakayama et al., 2006; Beaumont et al., 2007).

The relationship between these polymorphisms and the clinico-dermoscopic phenotypes of AMN are not well understood, and a better understanding may improve early detection and treatment strategies for melanoma. In addition, these polymorphisms are important in European individuals, but their effect on pigmentation and melanoma risk in other ethnic groups is largely unknown.

We hypothesize that the clinical and dermoscopic features of AMN are modulated by genetic variants. This study aimed to describe dermoscopic characteristics of AMN and *MC1R* polymorphisms in the Chinese Han population, and evaluate potential associations between dermoscopic naevus patterns, *MC1R* genotypes and clinical features of AMN.

## Materials and Methods

### Participant enrolment

The Bioscientific Ethics Committee of Zhengzhou University approved this study and all participants provided written informed consent. Inclusion criteria were Han ethnicity and the presence of AMN, diagnosed by two experienced dermatologists based on digital clinical and dermoscopic images of lesions, or atypical or equivocal lesions with a diagnosis of AMN confirmed by histopathology. Exclusion criteria were: current pregnancy; a history of UVA or UVB therapy, solar salon tanning, or intensive sun exposure; long term systemic administration of corticosteroids or immunosuppressants; having AMN which had a recent history of being traumatized or treated with topical caustics, cryotherapy, laser therapy, electronic surgical procedures, or surgical excision; or having AMN located within the lesions of other skin conditions. One hundred and fifty-seven participants were enrolled consecutively from 1 March 2013 to 30 September 2013 from patients presenting at the Inpatient Department of Dermatology, the First Affiliated Hospital, Zhengzhou University, Zhengzhou, China. This sample size was determined by the number of eligible participants presenting at the department. Forty-two patients invited to participate were subsequently deemed ineligible: eight declined to participate, five were physically unable to participate in the data collection, 14 had no AMN, two had AMN within the lesions of other skin conditions, two had AMN affected by topical caustics, surgical procedures or other trauma, and 11 had abnormal skin at the designated sites for measuring melanin. Authors retained information to identify individual participants during data analysis

### Clinico-dermoscopic data collection

For each participant, researchers recorded their demographic characteristics, sun protection approaches over the past five years, medical history, melanin values for the flexor and extensor forearm, skin tanning types, freckling features, hair and eye colour, and the clinical and dermoscopic features of AMN. Melanin values were measured on an absorption scale of 0 (lightest) to 99 (darkest) with a Skin Tone Pen TP 20 pigmentation probe (Courage + Khazaka Electronic Co Ltd., Cologne, Germany). Digital clinical and dermoscopic images of every suspected AMN ⩾2 mm in each participant were also recorded for further evaluation by experienced researchers. Clinical images of the AMN were obtained using a Cyber-shot DSC-F707 digital camera (Sony Corp, Tokyo, Japan). Dermoscopic images were acquired using a DermLite FOTO dermoscope (3 Gen, LLC, San Juan Capistrano, CA, USA) coupled with the same digital camera.

**Figure 1:**
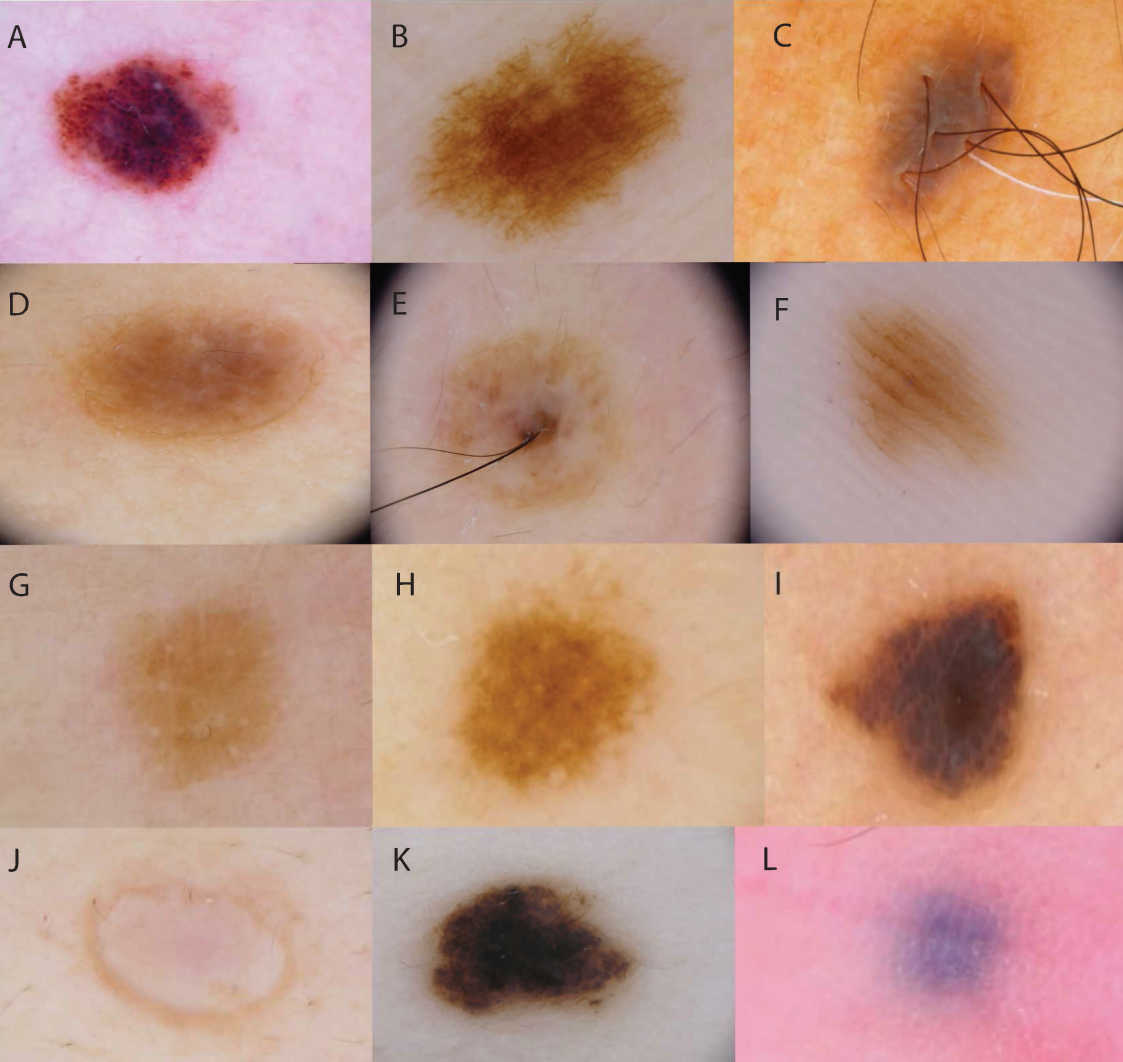
Examples of dermoscopic patterns and colours of AMN. Dermoscopic patterns are (A) globular, with round-to-oval pigmented structures; (B) reticular, with a net of lines; (C) homogenous, with uniform pigmentation and no pigment structures; (D, E) non-specific, with widespread structures that are neither globular nor reticular, OR chaotic with 2+ colours; (F) parallel furrow, with parallel lines of pigment. Colours classifications were (G) light brown, (H) brown, (I) dark brown, (J) flesh-coloured, (K) black, (L) blue.

Four hundred and forty-eight AMN lesions were chosen for dermoscopic assessment. All naevi ⩾2 mm in diameter were counted. The images were independently evaluated by two dermoscopists for dermoscopic global features, local features, dermoscopic subtype and predominant dermoscopic pattern, adapted from Argenziano and colleagues (Argenziano et al., 2007). The body sites of the AMN were recorded as head/neck, trunk, and limbs. We catalogued AMN into dermoscopic groups (globular, reticular, homogeneous and parallel furrow; naevi that did not show any of these morphological criteria were classified as nonspecific; see Fig. 1). The signature pattern of each patient was defined as the dermoscopic pattern of at least one-third of the patient’s lesions. Congenital naevi were excluded by asking participants which, if any, of their naevi were present between birth and 6 months of age. Histopathology was not performed on the naevi, as they were clinically benign and the risks posed by excision for histopathological analysis, such as scarring and potential infection, were considered excessive for an observational study of the clinical and dermoscopic characteristics of naevi.

Researchers also recorded the following clinical phenotypic characteristics: Fitzpatrick skin type (I–VI) (Fitzpatrick, 1988), freckling score ranging from one (none) to four (severe), hair colour (red/auburn, fair/blond, light brown, dark brown, or black), and eye colour (blue/grey, green/hazel, or brown) (Palmer et al., 2000; Duffy et al., 2004).

### DNA collection and sequencing

Blood samples were collected from 115 participants. DNA was successfully extracted from 111 samples with a DNA extraction kit (CW Bio, Inc., Peking, China) in the Key-Disciplines Laboratory, Clinical-Medicine Henan, the First Affiliated Hospital of Zhengzhou University, Zhengzhou, China. DNA quality was assessed at the Shenzhen branch of Beijing Genomics Institute, and 98 DNA samples were of sufficient quality to be sequenced at the *MC1R* gene. Nested PCR sequencing of *MC1R* was performed, using the following primer sets (Yamaguchi et al., 2012): Set 1: f 5′-CGAAATGTCCTGGGGACCTG-3′, r 5′-AACGGGGACCAGGGAGGTAA-3′. Set 2: f 5′-CAGGACACCTGGAGGGGAAG-3′, r 5′-CCCAGGGTCACACAGGAACC-3′. Sequencing primers: r 5′-CAGAGGCTGGACAGCATGGA-3′, r 5′-GTGAGGGTGACAGCGCCTTT-3′, f 5′-GGCCAGTGTCGTCTTCAGCA-3′.

### Genotyping

*MC1R* genotypes were analysed by the Dermatology Research Centre at The University of Queensland using the Sequencher sequence alignment program (Gene Codes Corporation, USA). Wild type was designated “wt”; D84E, R142H, R151C, R160W and D294H variants were classified as “*R*”; V60L, V92M, R163Q, I120T, R67Q, R233W, D184N, T308M and S172I were classified as “*r*” s (Duffy et al., 2004; Baron et al., 2014).

### Statistical analysis

We tested for correlations between *MC1R* genotype, clinico-dermoscopic features of AMN, freckling on the face and the melanin value of flexural upper arm with the Kruskal–Wallis test. *P* values <0.05 were considered significant.

## Results

### Patient characteristics

One hundred and fifteen AMN patients met the criteria for clinical analysis (79 patients with AMN ⩾ 2 mm, 115 patients with AMN < 2 mm), with 49 (42.6%) males and 66 (57.4%) females. The mean age of the patients was 38.2 years (range from 5.5 to 83). The total naevus count of females was 1,047 (15.9 lesions per person), while the total naevus count of males was 638 (13.0 lesions per person). For AMN ⩾ 2 mm, there were 258 lesions in 47 females (5.5 lesions per person), and 190 lesions in 32 males (5.9 lesions per person). All patients had a Fitzpatrick skin type III or IV, with 46 (40.0%) type III and 69 (60.0%) type IV. Twenty patients had freckles; facial, hand and shoulder freckling scores are shown in Table 1. The median melanin value of the flexor upper arm was 44.15, while the extensor forearm was 54.30. The melanin values of these participants’ arms are shown in Table 1. Hair colour of this population was varied: five (4.3%) were light brown, 49 (42.6%) were dark brown and 61 (53.0%) were black. All patients had brown eyes.

**Table 1:** Freckling score and melanin value of AMN patients.

	**Min**	**Mean**	**Max**
Facial freckling score	1	1.3	4
Hand freckling score	1	1	1
Shoulder freckling score	1	1	1
Melanin value flexor upper arm	19.00	45.43	99.00
Melanin value extensor forearm	20.00	56.19	99.00

### Clinical characteristics of AMN

**Figure 2:**
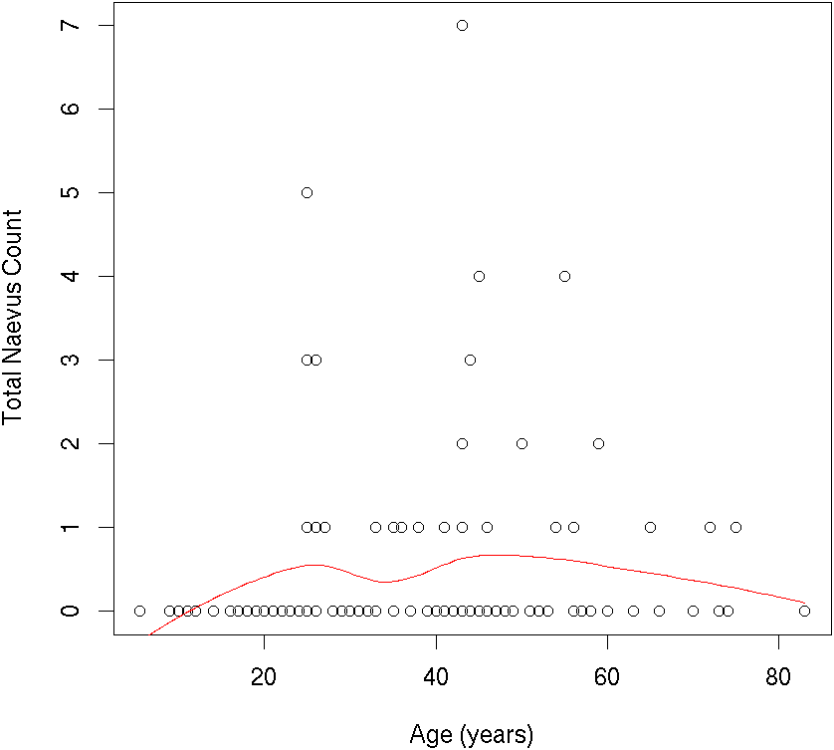
Individual participants’ ≥2 mm nevus count by age.

In our population, naevus number increased from childhood to early adulthood, and decreased from the mid-40s onward. Total naevus ⩾2 mm count by age is shown in Fig. 2. One thousand two hundred and thirty-seven AMN were <2 mm in diameter, and 448 were ⩾2 mm. Of the lesions ⩾2 mm, most were located on the head/neck (39.1%) and trunk (37.9%), with relatively few located on the limbs (23.0%). Most AMN ⩾2 mm were either light brown (53.8%) or dark brown (32.8%); however, there were also examples of black (6.3%), flesh-coloured (5.4%) or blue (0.9%) AMN and others (0.9%). The majority of AMN ⩾2 mm were macular in shape (70.8%); a large minority were a mildly elevated plaque (21.4%) or a markedly elevated plaque (7.8%). Most AMN (81.0%) had a smooth surface, while some had a coarse 12.3% or papillomatous (6.7%) surface. A small number of patients (5.1%) reported that one or more lesions were sometimes itchy. Clinical characteristics of AMN ⩾2 mm are shown in Table 2.

**Table 2:** Clinical characteristics of AMN.

**Characteristic**	**Patients *N* (%)**	**Lesions *N* (%)**
**Diameter of AMN**		
• <2 mm	115 (100.0)	1,237 (73.4)
•⩾2 mm	79 (68.7)	448 (26.6)
**Anatomic distribution of AMN ≥ 2 mm**		
• Head/Neck	57 (72.2)	175 (39.1)
• Trunk	64 (81.0)	170 (37.9)
• Limbs	43 (54.4)	103 (23.0)
**Color of AMN ≥ 2 mm**		
• Flesh-colored	20 (25.3)	24 (5.4)
• Light brown	66 (83.5)	241 (53.8)
• Dark brown	53 (67.1)	147 (32.8)
• Black	23 (29.1)	28 (6.3)
• Blue	4 (5.1)	4 (0.9)
• Others	3 (3.8)	4 (0.9)
**Shape of AMN ≥ 2 mm**		
• Macule	70 (88.6)	317 (70.8)
• Mildly elevated plaque	43 (54.4)	96 (21.4)
• Markedly elevated plaque	20 (25.3)	35 (7.8)
• Smooth	76 (96.2)	363 (80.1)
• Coarse (but not papillomatous)	35 (44.3)	55 (12.3)
• Papillomatous	18 (22.8)	30 (6.7)
**Symptom of AMN ≥ 2 mm**		
• No symptoms	75 (94.9)	443 (98.9)
• Sometimes itching	4 (5.1)	5 (1.1)

### Dermoscopic features

**Figure 3:**
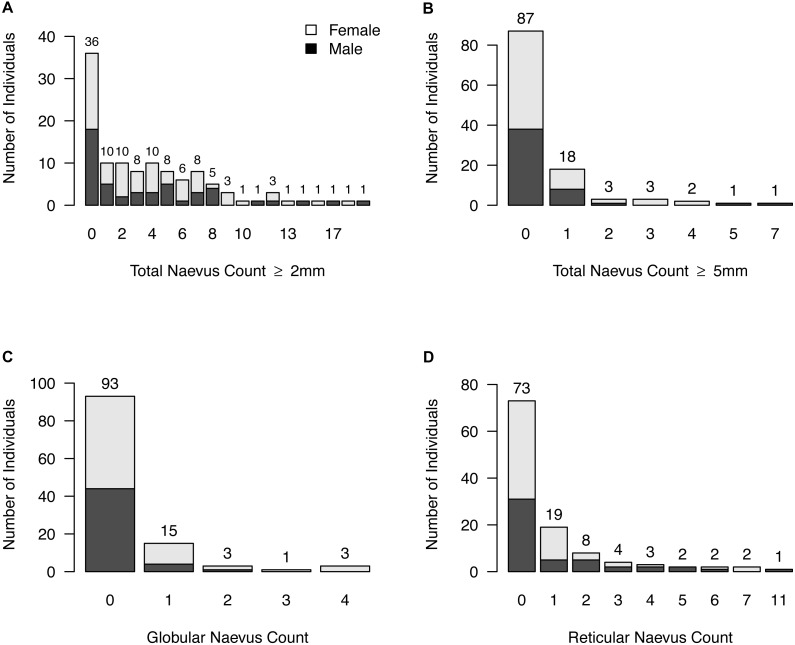
Naevus characteristics. (A) Frequencies of participants with same total neavus ≥2 mm count; (B) frequencies of participants with same total neavus ≥5 mm count; (C) frequencies of participants with same globular neavus count; (D) frequencies of participants with same reticular neavus count.

Of a total of 448 AMN ⩾2 mm, 309 lesions (69.0%) were nonspecific pattern, 105 (23.4%) were reticular, 33 (7.4%) were globular, one (0.2%) was parallel furrow. Dermoscopic characteristics are shown in Table 3 and Fig. 3.

 The signature dermoscopic pattern was nonspecific for 46 participants (58.2%), reticular for five participants (6.3%), a combined nonspecific-globular pattern for seven participants (8.9%), a combined nonspecific-reticular pattern for 20 participants (25.3%), globular for one participants (1.3%), and parallel furrow for one participant (1.3%). No signature pattern was assigned to the 36 participants who had no naevi ⩾2 mm.

**Table 3:** Clinico-dermoscopic phenotype characteristics.

**Phenotype**	**Min**	**Q1**	**Median**	**Mean**	**Q3**	**Max**
**AMN ≥ 2 mm**						
Total	0	0	0	1.5	2.5	15
Globular	0	0	0	0.3	0	4
Reticular	0	0	0	0.9	1	11
Nonspecific	0	0	2	2.7	4	18
**AMN ≥ 5 mm**						
Total	0	0	0	0.5	0	7
Globular	0	0	0	0.1	0	3
Reticular	0	0	0	0.2	0	7
Nonspecific	0	0	0	1.1	2	11

### Other features

Histopathology was not performed on these naevi, so it is not possible to accurately divide all naevi surveyed into histopathological categories such as junctional, compound or dysplastic naevi. However, 133 naevi (45 of these ⩾5 mm) were clinically elevated and therefore likely to correspond histopathologically to dermal naevi.

### *MC1R* polymorphisms

**Figure 4:**
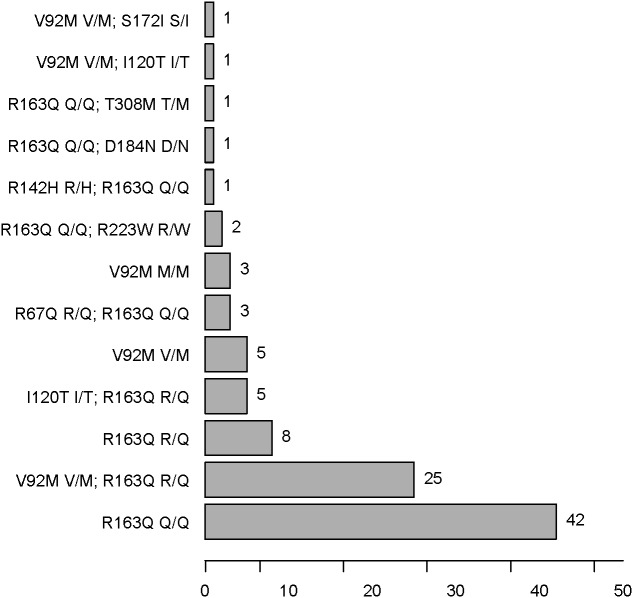
*MC1R* SNP frequencies in AMN patients.

There were four kinds of *MC1R* genotypes in our population: 77 *r/r*, seven *r*∕*r*∕*r* , one *R*∕*r*∕*r* , and 13 *wt/r* (see Fig. 4 and Tables 4 and 5). *R*∕*r*∕*r* and *r*∕*r*∕*r* indicate participants with two variants on one allele and a single variant on the other. The only patient with an *R* (R142H R/H) allele plus *r* alleles (R163Q Q/Q) has light brown hair, which is very rare in the Han population. Two other participants have light brown hair, with *r/r* genotypes (one R163Q Q/Q, the other V92M V/M and R163Q R/Q).

**Table 4:** *MC1R* genotypes detected.

**Category**	**Variants present**	**Patients *N* (%)**
***r/r***	R163Q Q/Q	42 (42.9)
	V92M V/M and R163Q R/Q	25 (25.5)
	I120T I/T and R163Q R/Q	5 (5.1)
	V92M M/M	3 (3.1)
	V92M V/M and I120T I/T	1 (1.0)
	V92M V/M and S172I S/I	1 (1.0)
***r/wt***	R163Q R/Q	8 (8.2)
	V92M V/M	5 (5.1)
***r/r/r***^a^	R67Q R/Q and R163Q Q/Q	3 (3.1)
	R163Q Q/Q and R223W R/W	2 (2.0)
	R163Q Q/Q and D184N D/N	1 (1.0)
	R163Q Q/Q and T308M T/M	1 (1.0)
***R/r/r***^a^	R142H R/H and R163Q Q/Q	1 (1.0)

**Notes.**

a These participants carried one allele containing two variants, with a single variant in the other allele.

**Table 5:** *MC1R* alleles detected.

**Category**	**rs number**	**Variant name**	**Variant allele**	**Red hair association^b^**	**Allele *N* (%)**
*wt*	N/A	Ancestral^a^	N/A	No	13 (6.6)
*R*	rs11547464^a^G/A	R142H	H	Strong	1 (0.5)
*r*	rs885479^a^G/A	R163Q	Q	Weak	138 (70.4)
	rs2228479^a^G/A	V92M	M	Weak	38 (19.4)
	rs33932559^a^T/C	I120T	T	No	6 (3.1)
	rs34090186^a^G/A	R67Q	Q	No	3 (1.5)
	rs372152373^a^C/T	R223W	W	No	2 (1.0)
	rs376670171^a^G/T	S172I	I	No	1 (0.5)
	rs530102853^a^G/A	D184N	N	No	1 (0.5)
	rs375127718^a^C/T	T308M	M	No	1 (0.5)

**Notes.**

a African allele as defined by Nakayama et al. (2006).

b In Europeans.

### Association between *MC1R* polymorphisms and phenotypic characteristics

**Figure 5:**
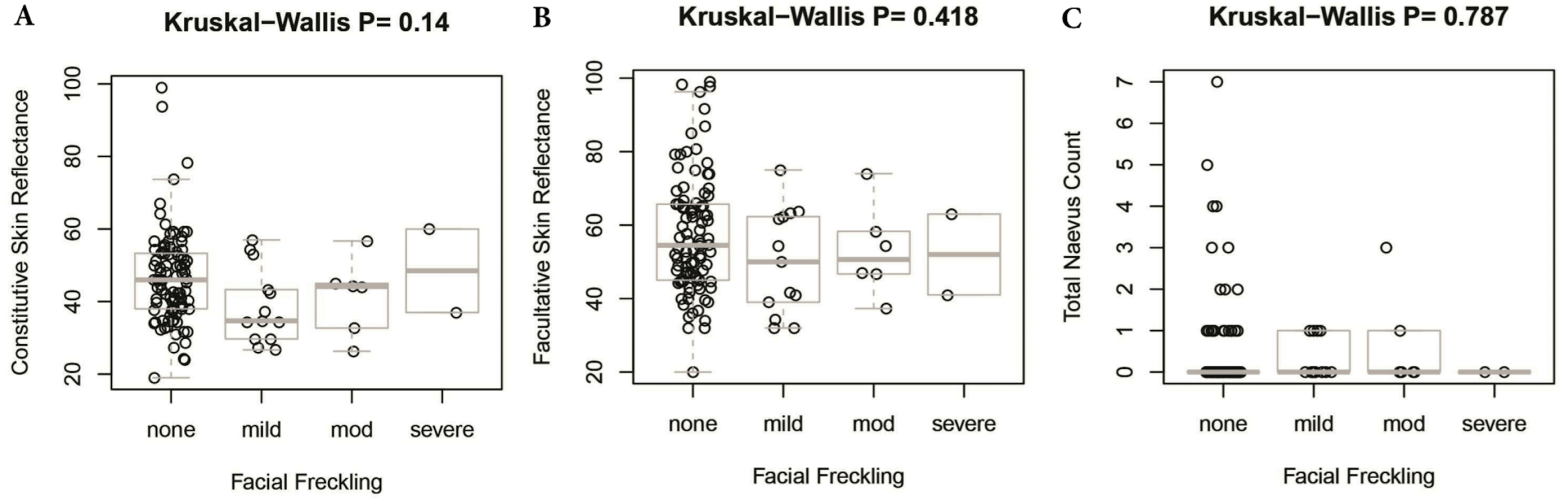
Association between facial freckling and skin reflectance or total nevus ≥2 mm count. (A) Association between facial freckling and constitutive skin reflectance; (B) association between facial freckling and facultative skin reflectance; (C) association between facial freckling and total neavus ≥2 mm count.

**Figure 6:**
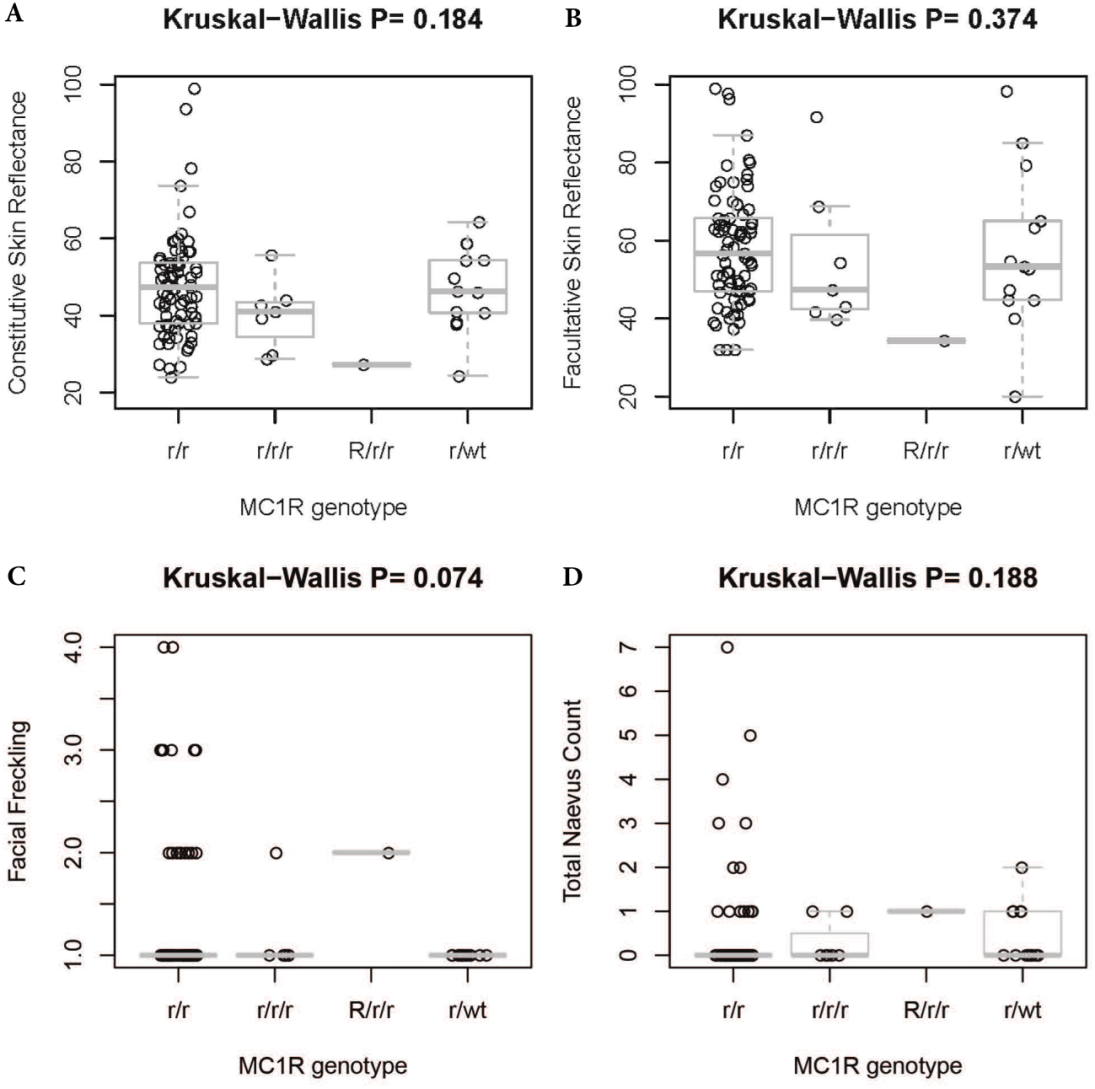
Association between *MC1R* genotype and skin reflectance, facial freckling, or total nevus ≥2 mm count. (A) Association between MC1R genotype and constitutive skin reflectance; (B) association between MC1R genotype and facultative skin reflectance; (C) association between MC1R genotype and facial freckling; (D) association between MC1R genotype and total neavus ≥2 mm count. *R/r/r* and *r/r/r* indicate participants with two variants on one allele and a single variant on the other.

**Figure 7:**
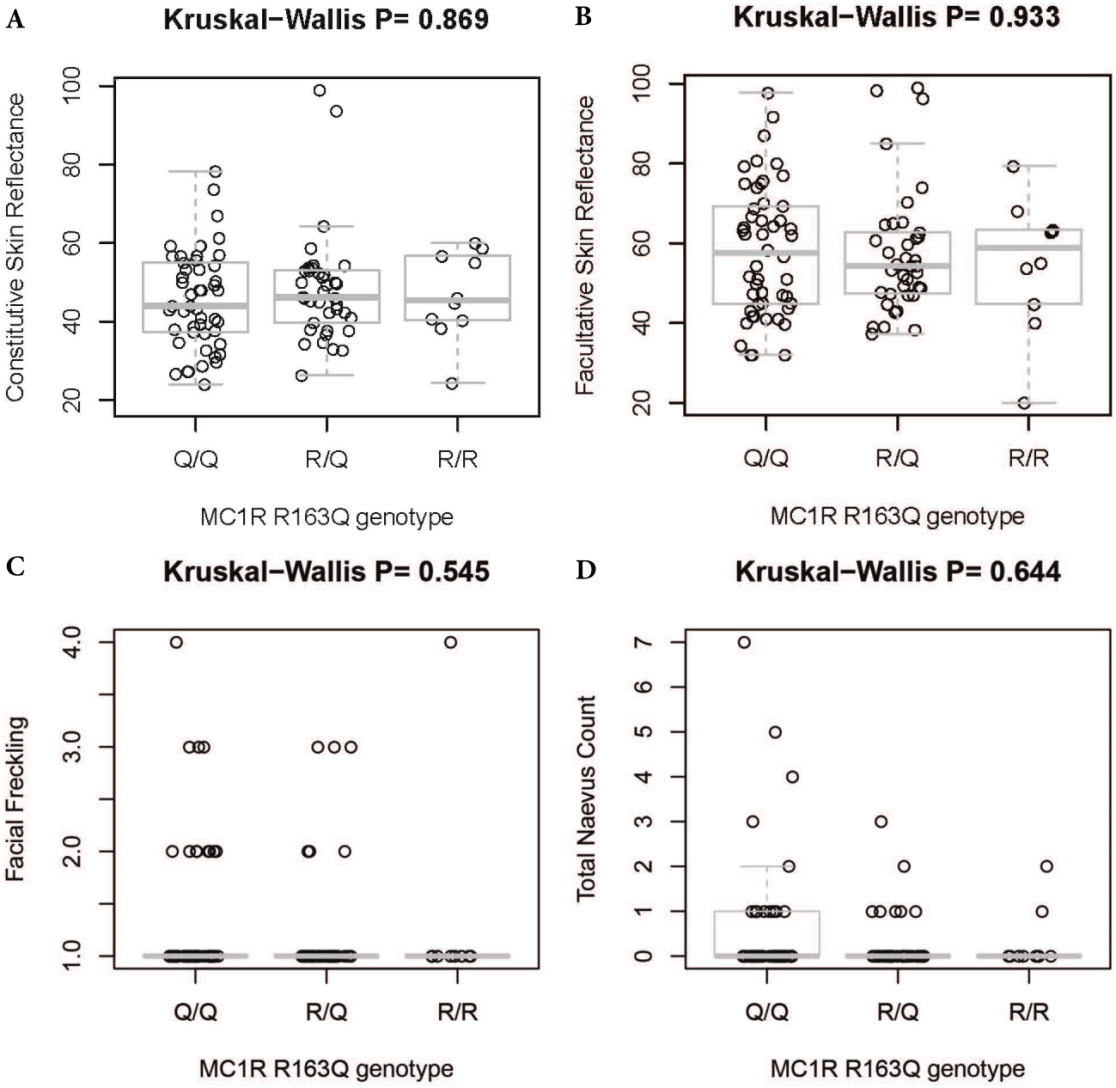
Association between *MC1R* R163Q genotype and skin reflectance, facial freckling, and total nevus ≥2 mm count. (A) Association between MC1R R163Q genotype and constitutive skin reflectance; (B) association between MC1R R163Q genotype and facultative skin reflectance; (C) association between MC1R R163Q genotype and facial freckling; (D) association between MC1R R163Q genotype and total neavus ≥2 mm count.

There was no significant difference in facial freckling between different groups of skin reflectance (constitutive skin reflectance *P* = 0.14, facultative skin reflectance *P* = 0.418). There was also no association between facial freckling and total naevus ⩾2 mm count (*P* = 0.787) or *MC1R* genotype (*P* = 0.074). There was no association between *MC1R* genotype and skin reflectance (constitutive skin reflectance *P* = 0.184, facultative skin reflectance *P* = 0.374) or total naevus ⩾2 mm count (*P* = 0.188). The *MC1R* variant R163Q was particularly examined, but no association was found between this variant and facial freckling (*P* = 0.545), skin reflectance (constitutive skin reflectance *P* = 0.869, facultative skin reflectance *P* = 0.933), or total naevus ⩾2 mm count (*P* = 0.644) (Figs. 5–7; Table 4).

## Discussion

In this study, we observed the clinical and dermoscopic characteristics of 115 AMN patients of Han ethnicity, where a low freckling score, brown or black hair and brown eye colour are common. In our sample, Han males have more naevi ⩾2 mm than Han females, while females have more naevi <2 mm than males. However, with a larger sample size, these differences may not persist.

Total naevus ⩾2 mm count peaked between 15 and 45 years of age in our study. This is consistent with studies of naevus number by age in patients from Australia, Austria, France, Italy, Spain and the US, which showed that naevus counts begin to increase in childhood, and decrease after the 4th decade of life (Zalaudek et al., 2011; Lee et al., 2016).

The majority of naevi in our study were found on the head, neck and trunk, contrasting with a previous study of naevi in 13–14 year old Italian children, which suggested that the best predictor of total naevus count was the lateral arms (Gallus et al., 2007). This difference may be due to our relatively small sample size or the effect of a different ethnic background. Since our population was mostly older than the Italian study, a higher cumulative sun exposure on the head and neck may also have influenced naevus distribution.

AMN ⩾2 mm in diameter in our Han population were predominantly brown or dark brown (86.6%), and macules or mildly elevated plaques in profile (92.2%). 69.0% of naevi examined had a nonspecific dermoscopic pattern, and 92.4% of individuals had a nonspecific signature pattern or a combined nonspecific-reticular/nonspecific-globular pattern.

The majority of naevi in our population were found in participants less than 60 years old, similar to previous research. The most common dermoscopic pattern in our study was nonspecific, followed by reticular, then globular pattern. Lee et al. (2016) found that globular naevi are more common in those younger than 15 years, and reticular naevi more common in those older than 15 years (Zalaudek et al., 2011). The same study also found a significant difference in the number of nonspecific naevi between monozygotic and dizygotic twins, suggesting that genetics plays a role in the formation of nonspecific naevi (Zalaudek et al., 2011). The predominance of the nonspecific dermoscopic pattern in the Han population is probably the result of a specific genotype.

We did not find a correlation between *MC1R* genotype and hair colour; however, previous studies have demonstrated that *MC1R R/R* and *R/r* variants are associated with red hair, lighter skin, green eyes and high facial freckling scores in Europeans. The R163Q variant, weakly relevant to red hair in Europeans (Raimondi et al., 2008), and V92M are very common in our AMN patients without a red hair phenotype, as expected from previous studies of *MC1R* in Asians (Nakayama et al., 2006).

There was no significant association between the R163Q variant and facial freckling in our study; in addition, hand and shoulder freckles were completely absent in our population. An investigation of *MC1R* polymorphisms, skin reflectance and freckles in a Japanese population showed that R163Q and V92M variants were significantly associated with increased odds of freckling (Yamaguchi et al., 2012). However, our results agree with an investigation of *MC1R* polymorphisms and freckling in a Chinese Han population from Chengdu, which found no association between V92M or R163Q and freckling (Cao et al., 2013). Bastiaens et al. (2001) found that nearly all individuals with freckles carried at least one *MC1R* variant, but half of those carrying *MC1R* variants did not express freckles. They estimated that the population-attributable risk for freckles to *MC1R* variants is 60% (Bastiaens et al., 2001). Our study also found no association between facial freckling and skin reflectance, or facial freckling and total naevus count.

Previous research on heritability of naevi and risk of melanoma indicates that total naevus count, changes in total naevus count, number of naevi 5.0–7.9 mm, and the presence of the nonspecific dermoscopic pattern should be considered by naevus surveillance programs (Lee et al., 2016). *MC1R* variants may be involved in melanoma development, but melanomas are not dependent on the classical RHC phenotype (Williams et al., 2011). Our results, showing no association between *MC1R* R163Q variant and skin reflectance, facial freckling or total naevus count, were not unexpected.

## Conclusions

In conclusion, we studied the pigmentation phenotype and naevus phenotype including dermoscopic pattern in a cohort of 115 Han Chinese from the Henan Province in China. In 98 of 115 study participants we analysed the *MC1R* genotype and correlated the findings with our phenotypic data. The correlation between pigmentation phenotype and *MC1R* genotype is in keeping with previous published findings. Not surprisingly there were no associations between *MC1R* polymorphisms and clinical or dermoscopic features of AMN in this Han Chinese cohort. In our estimation, further genomic work-up of this well-annotated cohort of Han Chinese will allow insights into candidate genes for naevus development.

##  Supplemental Information

10.7717/peerj.4168/supp-1 Supplemental Information 1 Phenotype data

10.7717/peerj.4168/supp-2 Supplemental Information 2 *MC1R* genotype results

## Additional Information and Declarations

### Data Availability

The following information was supplied regarding data availability: Lee, Katie (2017): Acquired melanocytic naevi in Han Chinese volunteers from Henan, China. figshare.
https://doi.org/10.6084/m9.figshare.4986143.
